# Editorial: Computational and experimental insights in proton and ion translocating bioenergetic systems

**DOI:** 10.3389/fchem.2024.1384385

**Published:** 2024-03-05

**Authors:** Vivek Sharma, Petra Hellwig, Manuela Pereira

**Affiliations:** ^1^ Department of Physics, University of Helsinki, Helsinki, Finland; ^2^ Laboratoire de Bioelectrochimie et Spectroscopie, UMR, CMC, CNRS University of Strasbourg, Strasbourg, France; ^3^ Department of Chemistry and Biochemistry, Faculty of Sciences, University of Lisbon and BioISI - Biosystems and Integrative Sciences Institute, Lisbon, Portugal

**Keywords:** bioenergetics, mitochondrial dysfunction, electron transport chain, computer simulations, biophysics

As a follow up of the Research Topic–“Computational and Experimental Insights in Redox-Coupled Proton Pumping in Proteins”, this Research Topic showcases how different experimental and computational approaches can be exploited to understand the molecular mechanisms of bioenergetic systems (Wikström et al., 2023a).

**FIGURE 1:**
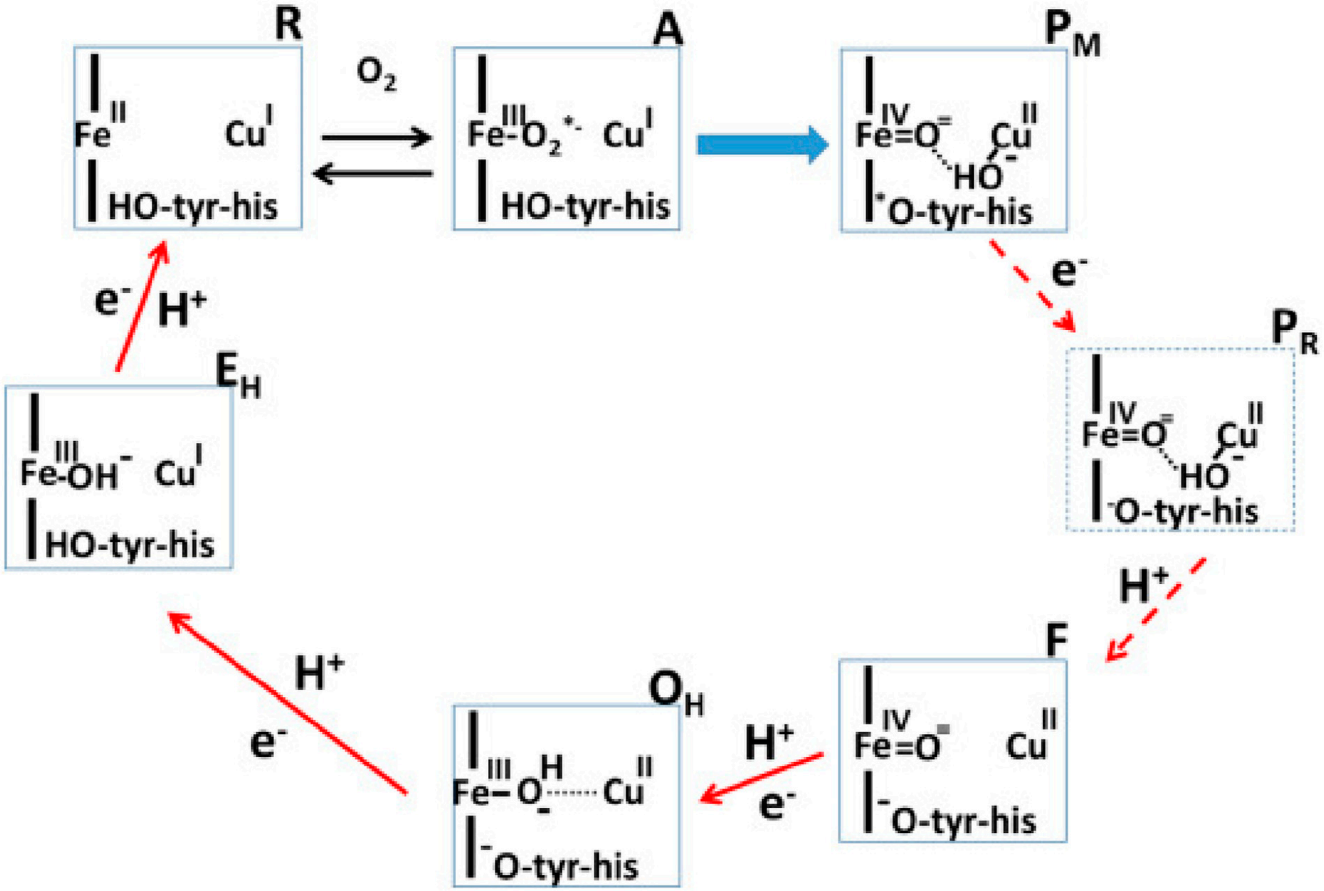
Catalytic cycle of A-type cytochrome *c* oxidase (Wikström et al., 2023a). Figure used with permission from the publisher.

Proteins performing redox-coupled proton pumping are key players in energy transduction in all living organisms. They are mostly observed in diverse respiratory chains (Marreiros et al., 2016; Calisto and Pereira, 2021). One of the most studied redox-coupled proton pumping proteins are oxygen reductases, the last enzymes of aerobic respiratory chains. These are found in all three domains of life, eukaryotes, prokaryotes and archaea and belong to the heme-copper oxidase superfamily or to the cytochrome *bd*-type family. The heme-copper oxidase superfamily has been divided primarily into three sub-types, A, B and C-type oxidases (Pereira et al., 2001). Mårten Wikström, in 1977, showed for the first time that mammalian cytochrome *c* oxidase, which belongs to type A, is a redox-driven proton pump (Wikström, 1977). We have come a long way since then and several aspects of its catalytic cycle are now well-understood (see Figure 1; Wikström et al. (2023b)).

In this Research Topic, Shimada et al. discuss the recent experimental findings which have significantly improved our understanding of the reaction mechanism of the mitochondrial enzyme. This is a contribution from Yoshikawa’s group that provided us with the breakthrough of the determination of the first structure of A-type mitochondrial cytochrome *c* oxidase (Tsukihara et al., 1995). There is convergence on several aspects of the catalytic cycle of oxidase, as discussed elegantly by Shimada et al. However, some central questions remain open–such as the role of the H-pathway of proton transfer in the proton pumping mechanism of A-type cytochrome *c* oxidase. Site directed mutagenesis studies, proton pumping experiments and computer simulation studies (Malkamäki et al., 2019; Maréchal et al., 2020; Reidelbach et al., 2021) suggest that H-pathway is not the proton pumping pathway in the yeast mitochondrial oxidase. Also, notably the exit route for pumped protons and the structure of the active site in resting oxidized state of the enzyme remains unclear. But new structural insights based on serial femtosecond X-ray crystallography studies by Ishigami et al. (2023) suggest that the conserved cross-linked tyrosine is neutral in the resting O state of the enzyme, in agreement with the proposals by Blomberg (Blomberg, 2021), whereas the origin of this proton maybe internal (Sharma and Wikström, 2016).

To obtain detailed molecular insights into enzyme mechanisms, computational approaches are often applied in combination with high resolution structural data (see Lee et al., 2022; Bergh et al., 2024). On one hand, physics based classical molecular dynamics (MD) simulations can help in providing high spatial and temporal resolution of biological processes, chemical reactions, on the other hand, can be studied with QM (Quantum Mechanics)-based computational approaches. Noodleman et al. discuss the dioxygen reduction reaction and proton pumping mechanism of B-type oxidases from *Thermus thermophilus*. They showcase the strength of computational approaches such as classical MD simulations and QM-based cluster calculations in understanding the mechanism of B-type oxygen reductase. The putative proton loading site, proton transfer pathways, exit route for pumped protons and active site intermediates of B-type oxidases, which share similarities and differences with the relatively well-understood A-type oxidases (Wikström and Sharma, 2019), are discussed.

Proton transfer reactions, which are central to redox-coupled proton pumping proteins have also been studied with hybrid QM/MM methods, combined with free energy analysis when required (see Mandal et al., 2023; Zdorevskyi et al., 2023). Protons can transfer rapidly along a hydrogen bonded pathway involving amino acid residues and water molecules. However, hydrogen bonds can be short-lived and undergo rearrangements during the catalytic cycle of a protein. This is the essence of the work by Bertalan and Bondar, who discuss graph-based approaches to study hydrogen bond networks in proteins with special focus on rhodopsins. Structural snapshots obtained from serial femtosecond crystallography were subjected to graph computations and hydrogen bond restructuring was resolved, giving novel insights into protein function. This work highlights integration of graph-based computational approaches to structural biology experiments with a possibility of extension also to MD simulation trajectories.

Cytochrome *bd*-type enzymes have found increasing interest in the last years. These enzymes are electrogenic and catalyze the reduction of molecular oxygen to water by recruiting protons from the cytoplasmic side of the membrane. However, they do not actively pump protons across, and in this way can be considered less efficient than heme-copper oxidases, which generate proton motive force both by pumping protons and recruiting substrate protons and electrons from the opposite sides of the membrane. Importantly, cytochrome *bd*-type oxidases are discussed to be defence factors in bacteria and are important antimicrobial drug targets (Friedrich et al., 2022). They turned out to show a high degree of diversity as shown by structural (Safarian et al., 2016; Safarian et al., 2019; Theßeling et al., 2019), electrochemical (Nikolaev et al., 2021) and also phylogenetic studies (Murali et al., 2021). The high diversity of these enzymes is confirmed with a new cryo-EM structure of cytochrome *bd* from *Corynebacterium glutamicum* (Grund et al.) that shares structural characteristics with the *Mycobacterium tuberculosis bd*-type enzyme.
